# Determining the insulin secretion potential for certain specific G-protein coupled receptors in MIN6 pancreatic beta cells

**DOI:** 10.3906/sag-1712-147

**Published:** 2019-02-11

**Authors:** Hasan GENÇOĞLU, Kazim ŞAHİN, Peter M. JONES

**Affiliations:** 1 Molecular Biology and Genetics Program, Department of Biology, Faculty of Science, Fırat University, Elazığ Turkey; 2 Department of Animal Nutrition and Nutritional Disorders, Faculty of Veterinary Medicine, Fırat University, Elazığ Turkey; 3 Division of Diabetes and Nutritional Sciences, Diabetes Research Group, King’s College London, London UK

**Keywords:** Pancreatic B cells, G-protein coupled receptors, MIN6, diabetes mellitus, cell culture

## Abstract

**Background/aim:**

The polypeptide hormone insulin is essential for the maintenance of whole-body fuel homeostasis, and defects in insulin secretion and/or action are associated with the development of type 1 and type 2 diabetes. The aim of this study was to assess the role of some G-protein coupled receptors (GPCRs), GPR54, GPR56, and GPR75, and cannabinoid receptors CB1R and CB2R, in the regulation of pancreatic β-cell function.

**Materials and methods:**

Insulin secretion from mouse insulinoma β-cell line (MIN6) monolayers was assessed via insulin radioimmunoassay (RIA). Reverse transcription-polymerase chain reaction (RT-PCR) was used to assess the expression of some specific GPCRs and the other receptors by MIN6 pancreatic β-cells.

**Results:**

The agonists were not found to be toxic for the MIN6 pancreatic β-cells within the range of the doses used in this study, whereas insulin secretion altered depending on the ligands and receptors. In addition, arachidonyl-2’-chloroethylamide (ACEA), carbachol, chemokine (C-C motif) ligand-5 (CCL5), and exendin as well as phorbol myristate acetate (PMA) ligands showed significant increases in the insulin secretion of MIN6 pancreatic β-cells.

**Conclusion:**

Understanding the normal β-cell function and identifying the defects in β-cell function that lead to the development of diabetes will generate new therapeutic targets.

## 1. Introduction

Type 2 diabetes (T2DM) is characterized by a combination of peripheral insulin resistance and inadequate pancreatic β-cell insulin secretion. By the year 2040, it is estimated that more than 642 million people worldwide will suffer from diabetes and nearly 90% of them will have T2DM (1). Poorly regulated T2DM eventually leads to a reduced life expectancy and quality of life as a result of secondary complications that cause a significant burden for both the affected individual and society at large (2,3). Current pancreatic β-cell targeted pharmacological therapies for T2DM have undesirable off-target effects and insufficient activity and tend to become less effective with disease progression, so there is an urgent need for novel therapeutic strategies (4). 

Insulin is synthesized and secreted exclusively by pancreatic β-cells located in the islets of Langerhans, which are aggregates of approximately 3000 endocrine cells that are found dispersed throughout the exocrine tissue pancreas (5). Mammalian islets are approximately spherical organs, with a median diameter of 100–200 µm, although a minority of islets may be much smaller (<50 µm) or larger (up to 500 µm) than this. The endocrine cells that form the islets of Langerhans are typical polypeptide hormone-secreting cells, with all the characteristics of such cells, including extensive rough endoplasmic reticulum, a well-developed Golgi system, and large numbers of membrane-bound vesicles in which the hormone is stored before being released by exocytosis (6). A typical islet comprises several thousand endocrine cells, including insulin-expressing β-cells (~60% of adult human islet cells), glucagon-expressing α-cells (20%–30%), somatostatin-expressing δ-cells (~10%), pancreatic polypeptide-expressing cells (<5%), and ghrelin-expressing cells (~1%). This cellular heterogeneity can lead to difficulties in interpreting studies using islets, so a variety of clonal hormone-secreting cell lines have been developed to allow the use of homogeneous populations (7). In this study, we have used the insulin-secreting MIN6 mouse insulinoma cell line as an experimental model (8,11). MIN6 cells synthesize and secrete insulin, and they can be used either as adherent monolayers (8) or as three-dimensional islet-like structures (pseudoislets) (9). Insulin secretion from pancreatic β-cells is regulated by both metabolic pathways (glucose, amino acids, nonesterified fatty acids) and nonnutrient extracellular signals (hormones, incretins, nonesterified fatty acids, neurotransmitters, and various small molecules) acting predominantly via G-protein coupled receptors (GPCRs) (8). Among the factors that reach the β-cell via the circulation, the incretin hormone glucagon-like peptide-1 (GLP-1) is of clinical relevance because it is a powerful enhancer of glucose-induced insulin secretion and has recently been introduced as a new therapy for T2DM (10). 

The aim of this study was to assess the roles of novel GPCRs in the regulation of β-cell function by measuring the effects of their activation on insulin secretion. The GPCR agonists chosen for the study were chemokine (C-C motif) ligand-5 (CCL5), which is a ligand for GPR75; arachidonyl-2’-chloroethylamide (ACEA), which is a cannabinoid receptor-1 (CB1R) agonist; carbachol (CCh), which is a muscarinic cholinergic agonist; and exenatide, which is a GLP-1 receptor agonist.

## 2. Materials and methods

### 2.1. Reagents

The pancreatic β-cells were maintained in a humidified atmosphere (37 °C; 95% air/5% CO2) in Dulbecco’s modified Eagle’s culture medium (DMEM), supplemented with 10% fetal bovine serum (FBS), 2 mM glutamine, 100 U/mL penicillin, and 100 µg/mL streptomycin) from Sigma-Aldrich (Dorset, UK). General laboratory chemicals, including carbachol, forskolin, and phorbol myristate acetate (PMA) were also from Sigma-Aldrich. The DMEM also contained phenol red, which acted as a pH indicator and allowed for a quick way of ensuring that the cells were growing in the appropriate environment; if the cells were at a too high a density or if the medium had not been changed regularly, there was a buildup of acidic metabolites and the medium changed from orange/red to yellow. The culture medium was changed every 3–4 days. QuantiTect SYBR Green PCR kits were obtained from QIAGEN (Manchester, UK). Primers that were used for standard PCR were prepared in-house (Molecular Biology Unit, King’s College London). Standard PCR was carried out using a Px2 Thermal RT-PCR cycler (Thermo Scientific, Epsom, UK). 

### 2.2. Cell culture and viability

For the experiments, the mouse insulinoma β-cell line MIN6 was used at low passage numbers (passages 29 to 40). The MIN6 pancreatic β-cell line was originally described in 1990 (11). This cell line was generated by the development of pancreatic insulinomas following microinjection of recombinant insulin/SV40 T antigen into the pronuclei of fertilized mouse eggs. The MIN6 cells used in these studies were kindly provided by Dr Y Oka and Professor JI Miyazaki (University of Osaka, Osaka, Japan). The viability of the cells during the insulin secretion experiments was evaluated with the trypan blue dye exclusion test, which is used to determine the number of viable cells in a cell suspension (12).

#### 2.2.1. Subculture and maintenance of MIN6 pancreatic β-cells

MIN6 pancreatic β-cells were cultured in negatively charged tissue culture flasks of 25 cm2 (T25) or 75 cm2 (T75), where they formed a monolayer upon seeding. 

The cells were maintained in a humidified atmosphere (37 °C; 95% air/5% CO2) in culture medium (DMEM) supplemented with 10% FBS, 2 mM glutamine, 100 U/mL penicillin, and 100 µg/mL streptomycin. 

Once the cells reached 60%–70% confluency they were subcultured to promote further propagation or for use in experiments. This process was carried out by trypsinization. 

A solution of trypsin/EDTA (0.1%/0.02%) from Sigma-Aldrich was used to detach the cells from the tissue culture plastic.

### 2.3. Static incubation measurements of insulin secretion

Adherent cells in 96-well plates were washed and preincubated in buffers containing a substimulatory concentration of glucose (2 mM) to establish a basal rate of insulin secretion. Cells were incubated (30, 60 min) with incubation buffer supplemented with secretagogues of interest (20 mM glucose, PMA, GPCR agonists, clonidine, exendin, etc.). Samples of the incubation medium were removed and stored at –20 °C until being assayed for hormone content. Cell viability was assessed by trypan blue exclusion under a light microscope. 

### 2.4. Radioimmunoassay

Dynamic insulin secretion of the MIN6 pancreatic β-cells was detected via an in-house radioimmunoassay (RIA) method based on the competitive binding to an insulin antibody of radiolabeled insulin and sample insulin. Standard samples were prepared with a serial dilution of 10 ng mL–1 insulin (Sigma) in borate buffer (133 mM boric acid, 68 mM NaOH, 10 mM EDTA (Sigma), 0.5 mg mL–1 BSA). Unknown samples were diluted in borate buffer to an expected insulin concentration of 0.5 ng mL–1, and then standards in triplicate and unknown samples in duplicate were added to LP3 tubes.

Iodine-125 (I125) labeled insulin was diluted to give a count per minute of 10,000 per 100 µL. Insulin primary antibody was derived from Hartley guinea pigs challenged with bovine insulin and 1:60,000 I125-labeled insulin and insulin antibody concentrations were added to all standards, samples, and relevant controls. 

After 48 h of incubation at 4 °C, γ-globulin solution (γ-globulin (Sigma), 30% PEG, Tween 20 (Sigma)) was used for precipitation. Following the precipitation, samples were read in a γ-counter (WIZARD2, PerkinElmer, Waltham, MA, USA).

### 2.5. Detection of mRNAs by RT-PCR

RNA was isolated from MIN6 cells and reverse-transcribed into cDNAs as described previously (13), and cDNAs were amplified using primers for the receptors. mRNA primers are shown in the Table. Amplicons were standardized against amplified actin mRNA, and control reactions replaced cDNA with water. SYBR Green Supermix was from Bio-Rad (Hercules, CA, USA). The PCR products were separated on 1.8% agarose gels, extracted using the QIAGEN Gel Extraction Kit, and visualized in the Molecular Biology Unit at King’s College London.

**Table 1:** Primers used for RT-PCR.

Gene	F/R*	Sequence
GPR75	F	5′-CCCTCACCATCATCCTCACT-3′
	R	5′-CCTTCGAGTGACAAACACGA-3′
GPR56	F	5′-GGCTGGAAATCCAGGAGGAC-3′
	R	5′-TCCAAACTGTGCTGCTTTGC-3′
GLP1R	F	5′-GTTTCGGAAATGCTGGGAGC-3′
	R	5′-GTAGGAACTCTGGCAGGTGG-3′
M3R	F	5′-GGGGAACTTAGCCTGTGACC-3′
	R	5′-AGAACAAGATGGCAGGAGCC-3′
CB1R	F	5′-GGAGCAGAGCAGGGGTTC-3′
	R	5′-AACCAACGGGGAGTTGTCTC-3′
ACTIN	F	5′-AGAGAAGATGACGCAGATAATGT-3′
	R	5′-GGTAAAGCTGTAGCCCCGTT-3′

*F: Forward; R: reverse.

### 2.6. Statistics

Student’s t-test, one-way ANOVA, and Tukey’s multiple comparison tests were used for evaluating the differences between means as appropriate and data were plotted using Prism software. Values of P < 0.05 were considered to be significant.

## 3. Results

### 3.1. MIN6 pancreatic β-cell culture 

**Figure 1:**
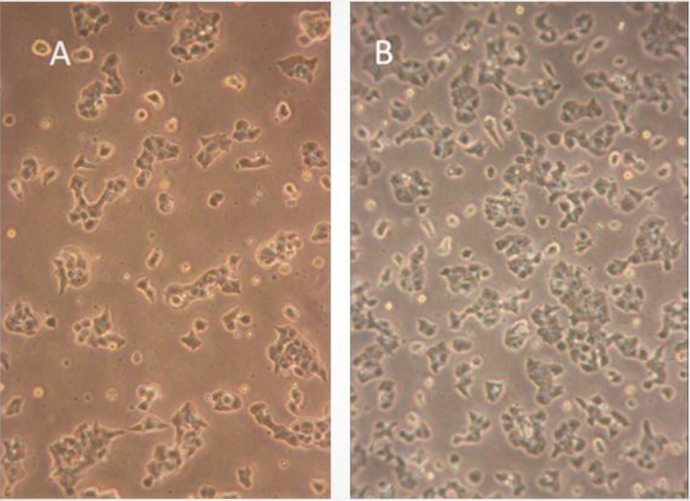
MIN6 pancreatic β-cells in culture. A) MIN6 pancreatic β-cells growing as approximately 30% confluent monolayer. B) The cells expand until they reach 60%–70% confluency, at which stage they are passaged or used for insulin secretion experiments.

Figure 1 shows two micrographs of MIN6 pancreatic β-cells growing as an adherent monolayer on tissue culture plastic. The morphology of the cells and trypan blue exclusion tests demonstrated that the cells were viable and healthy, and thus suitable for static incubation insulin secretion experiments. 

### 3.2. RT-PCR 

**Figure 2:**
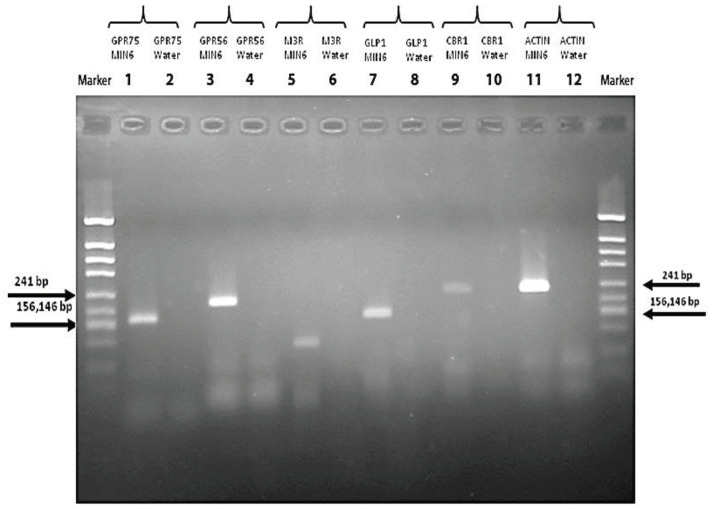
MIN6 pancreatic β-cell mRNA expressions of the receptors G-protein coupled receptor 75 (GPR75), G-protein coupled receptor 56 (GPR56), muscarinic acetylcholine receptor (M3R), glucagon-like peptide-1 receptor (GLP1R), cannabinoid receptor 1 (CB1R), and actin.

In the current study, selected receptor mRNA expressions of MIN6 pancreatic β-cells were examined by reverse transcription-polymerase chain reaction (RT-PCR). Figure 2 shows that MIN6 pancreatic β-cells express mRNAs for GPR75, GPR56, the GLP-1 receptor, and actin, with amplicons migrating at the appropriate size when compared to known standards (“Marker”). The expression of these receptors by the MIN6 pancreatic β-cells is consistent with the effects of their ligands on insulin secretion.

### 3.3. Glucose-stimulated insulin secretion (GSIS) in MIN6 pancreatic β-cells

**Figure 3:**
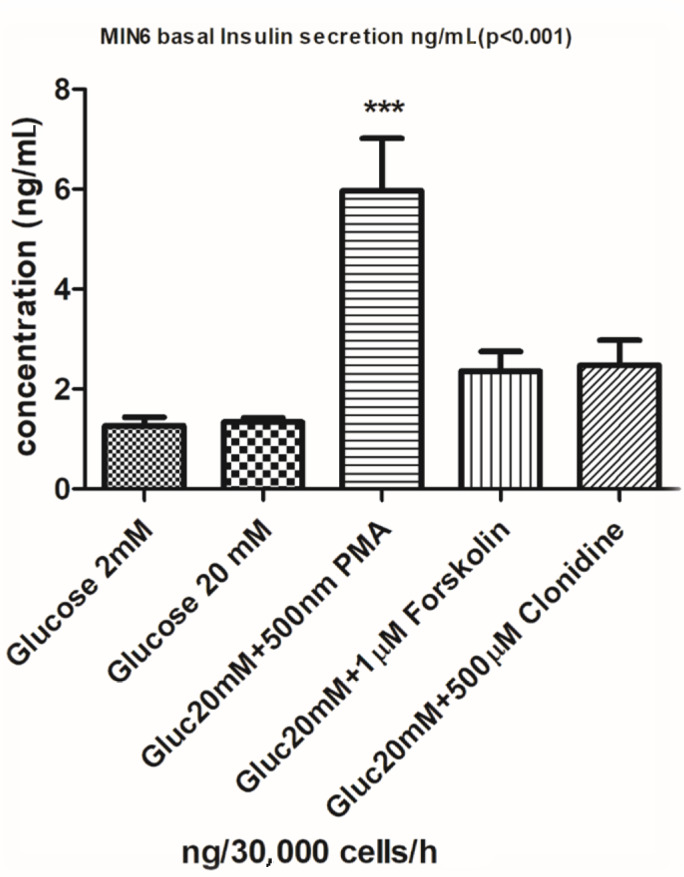
Insulin secretion analysis with phorbol myristate acetate (PMA), forskolin, and clonidine from MIN6 pancreatic β-cell monolayers. ***P < 0.001.

**Figure 4:**
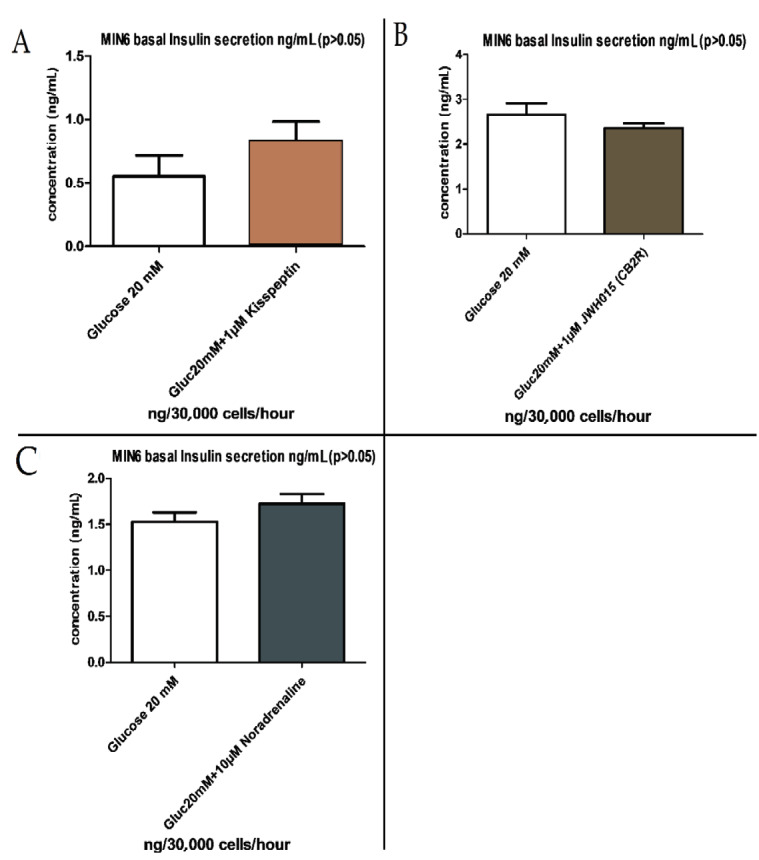
Insulin secretion analysis with A) kisspeptin (GPR54 agonist), B) JWH015 (CB2R agonist), and C) noradrenaline (adrenergic receptor agonist) from MIN6 pancreatic β-cell monolayers (P > 0.05).

Increasing the glucose concentration from 2 mM to 20 mM did not initiate a secretory response from the MIN6 pancreatic β-cells, which is in accordance with previous observations that this cells line loses responsiveness to glucose with later passages. However, the protein kinase C activator PMA induced a statistically significant insulin secretory response, and 1 mM of the adenylate cyclase activator forskolin and 500 mM of the alpha adrenoreceptor agonist clonidine increased the rate of secretion, albeit not to a significant extent in this experiment (Figure 3). Figures 4A–4C show that the GPR54 agonist kisspeptin, the CB2R agonist JWH015, and the adrenergic agonist noradrenaline had no significant effects on glucose-dependent (20 mM) insulin secretion from MIN6 cell monolayers. The observations using PMA demonstrate that the activators of protein kinases known to be involved in stimulus-secretion coupling in pancreatic β-cells could stimulate insulin secretion from MIN6 cells, therefore validating the use of the cells for future studies of insulin secretion.

### 3.4. Insulin secretion experiment results in target agonists and optimal dosages

**Figure 5:**
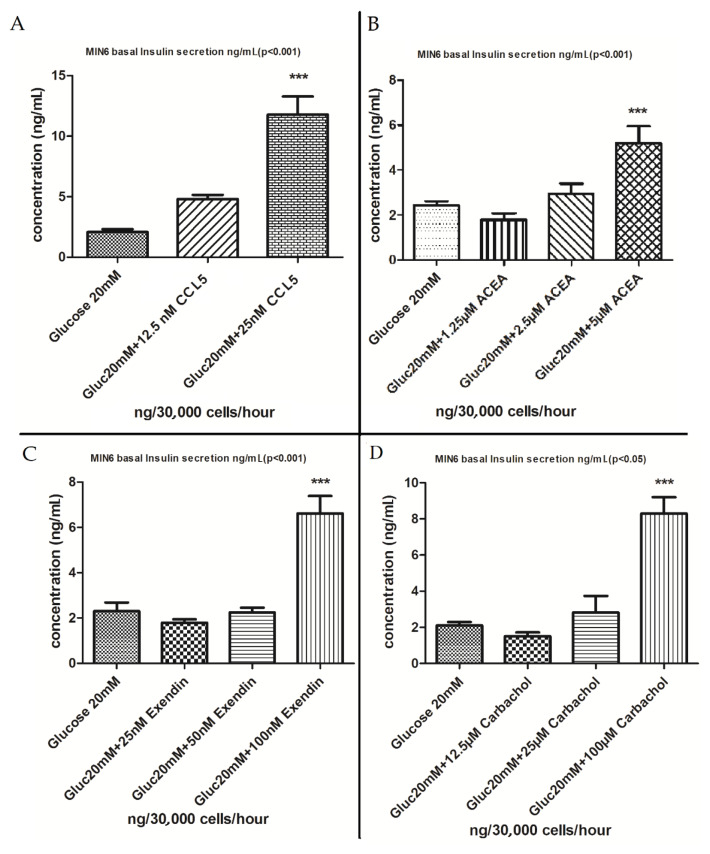
Insulin secretion analysis from MIN6 pancreatic β-cell monolayers to detect the optimal agonist dosages: A) CCL5 (GPR75 agonist), B) ACEA (CB1R agonist), C) exendin (GLP1R agonist), and D) carbachol (M3R agonist). ***P < 0.001.

Since the protein kinase C activator PMA induced a statistically significant insulin secretory response, it was subsequently used as a positive control for further studies. Preliminary studies identified CCL5 (GPCR75 agonist), ACEA (CBR1 agonist), exenatide (GLP1R agonist), and CCh (M3R agonist) as candidates for further study. Figures 5A–5D show the concentration-dependent effects of these agonists to significantly stimulate insulin secretion from MIN6 cell monolayers.

## 4. Discussion

Glucose-stimulated insulin secretion (GSIS) is a complex pathway involving glucose metabolism (glycolysis and mitochondrial oxidation), plasma membrane depolarization, calcium signaling, and exocytosis, taking place in pancreatic β-cells (14). Pancreatic β-cell disorders play an important role in the pathogenesis of both types of diabetes, and the resolution of the physiological behavior of these cells has a great influence on the prevention and treatment of this disease (15). Ligands that activate specific GPCRs in pancreatic β-cells have been reported to potentiate GSIS (16).

In this study, we focused on GPCR agonists and receptors including kisspeptin-1 receptor GPR54, adhesion type receptor GPR56, and orphan receptor GPR75, as well as some other agonists and receptors including cannabinoid receptors CB1R and CB2R, glucagon-like peptide-1 receptor (GLP1R), beta-adrenergic receptor noradrenaline, and muscarinic acetylcholine receptor (M3R), analyzed to determine the exact insulin secretory dosages for these receptors and ligands in MIN6 cells. Forskolin and PMA are well known to stimulate insulin secretion from β-cells through the activation of adenylate cyclase or protein kinase C, respectively (9,16,17). In our studies, PMA was a more effective stimulator than forskolin, so it was used as a positive control in subsequent experiments using GPCR agonists. Repeated measurements have been used to validate the insulin secretion studies, and repeated PCR measurements using actin as an internal standard mRNA were used to assess receptor expression. After PCR analysis of receptor expression, we chose several specific agonists to assess the effects of receptor activation on insulin secretion (kisspeptin, CCL5, ACEA, JWH015, exendin, noradrenaline, and CCh). 

Our insulin secretion data suggest that the GPR75 ligand CCL5, the CBR1 receptor agonist ACEA, the GLP1R agonist exendin, and the muscarinic receptor agonist CCh are the most effective receptor-operated ligands in terms of stimulating insulin secretion. In addition, the tumor-promoting protein kinase C activator PMA consistently enhanced insulin secretion, demonstrating the importance of the protein kinase C pathway in the regulation of insulin secretion (18). Although we found that MIN6 pancreatic β-cells express mRNA for the orphan receptor GPR56, we did not have the opportunity to assess the effects of collagen III, which has been reported to be a GPR56 ligand (19); thus, we have not evaluated its effects on insulin secretion in the current study.

It was reported that GPR75 is a receptor of chemokine (C-C motif) ligand-5 (CCL5) on the pancreatic β-cell membrane (20). It is involved in stimulating insulin secretion and improves glucose homeostasis in both lean mice and ob/ob insulin-resistant mice. Here we verified its ability to enhance insulin secretion from MIN6 pancreatic β-cells.

The RT-PCR results suggest that the amplicons for some receptors (e.g., M3R, CBR1) were less pronounced than others, although their ligands (Cch, ACEA) were effective in stimulating insulin secretion. There are two possible causes for these observations: the PCR analysis was not using a real-time quantification protocol, so the primers may show lower affinity when used in standard RT-PCR, as in the current study; or the agonists were interacting with other unidentified receptors to stimulate insulin secretion, although this seems unlikely given the known specificity of CCh for M3 muscarinic cholinergic receptors.

Because diabetic patients mainly have increased risks of cardiovascular morbidity and mortality, the comparative evaluations of the studies including GPCR agonists may improve not only myocardial function but also the overall quality of life in human patients (21,22).

 In conclusion, the present study indicated that different doses of the ligands and agonists were not toxic to MIN6 cells, whereas insulin secretions changed. In addition, gene expressions for some agonists (ACEA, carbachol) were not detected in mRNA receptor expressions (CBR1, M3R), but induced insulin secretion in the cells.

**Figure 6:**
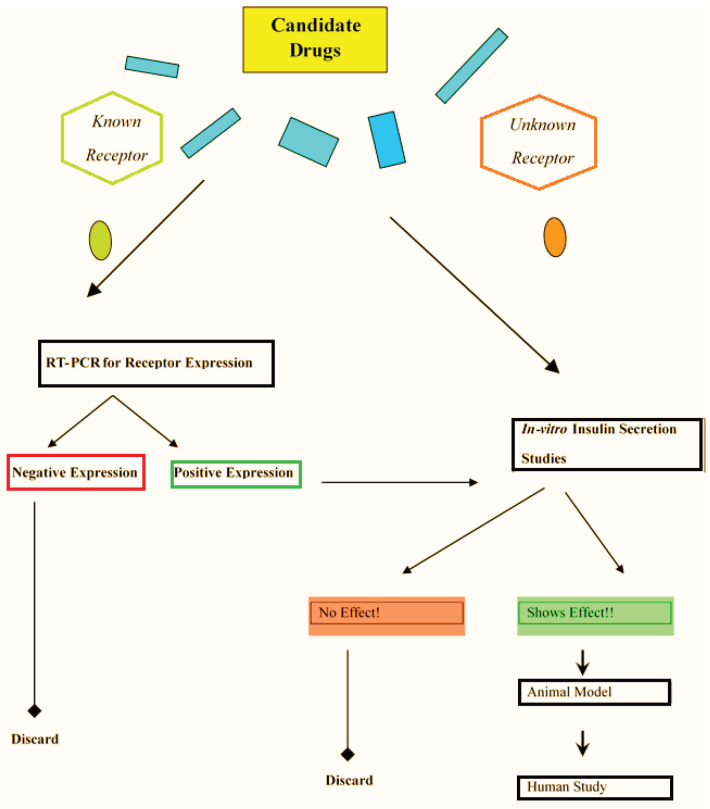
Drug discovery for agonist-receptor complexes in type 2 diabetes mellitus.

 Moreover, the ACEA, carbachol, CCL5, exendin, and PMA ligands showed significant increases in insulin secretion of MIN6 beta cells. Further studies including beta receptors’ communication in the treatment of diabetes mellitus as well as animal experiments involving the insulin secretion effect of their ligands are required (Figure 6). 

## Acknowledgments

Hasan Gençoğlu received financial support from the Scientific and Technological Research Council of Turkey (TÜBİTAK) BİDEB-2219 International Postdoctoral Research Fellowship Program (Project No: 1059B191300988) during this study. He also appreciates the kind support of the Diabetes Research Group at King’s College London, UK. This study was partially presented as an oral abstract at the 4th National Molecular Biology and Biotechnology Congress in Afyonkarahisar, Turkey.
